# When Silence Masks Autism: Delayed Autism Diagnosis in an Adolescent With Deafness

**DOI:** 10.1155/crps/8974477

**Published:** 2026-08-30

**Authors:** Stephanie E. King, Erika Ryst

**Affiliations:** ^1^ Department of Speech Pathology and Audiology, University of Nevada, Reno School of Medicine, Reno, Nevada, USA, unr.edu; ^2^ Nevada Center for Excellence in Disabilities, College of Education and Human Development, University of Nevada, Reno, Nevada, USA, unr.edu

**Keywords:** autism spectrum disorder, case report, deaf and hard of hearing, dual diagnosis, late diagnosis

## Abstract

A 13‐year‐old adolescent male was referred for an interdisciplinary autism assessment. His complex medical history included a traumatic delivery (with drop in heartrate and nuchal cord), neonatal hyperbilirubinemia, diagnosis of profound congenital hearing loss, diminished bile ducts on liver biopsy at 1 month of age, and a seizure disorder. Later, he was diagnosed with a significant global developmental delay with minimal communication in any modality. The youth received cochlear implants at the age of 2 years but was unable to tolerate them. Despite early intervention services and multiple stays in urban academic medical centers, he was not considered for a diagnosis of autism because his problematic behaviors, sensory sensitivities, and social and communication delays were attributed to his hearing loss and intellectual disability. It was not until the age of 13 that he was confirmed to have autism via a comprehensive interdisciplinary assessment based on adapted, standardized, evidence‐based tools and expert clinical observations. This case highlights the importance of understanding and identifying the differences between hearing loss related behaviors and possible symptoms of a co‐occurring neurodevelopmental disorder as well as the peril of diagnostic overshadowing in children with hearing loss.

## 1. Introduction

Autism spectrum disorder (ASD) is a neurodevelopmental condition characterized by (1) deficits in the social use of language and (2) unusual, repetitive, and fixed behaviors and interests. The Center for Disease Control currently estimates that one in 31 U.S. children have been identified with an ASD [1]. ASD is a complex disorder with variable presentations that make it difficult to diagnose; in fact, despite proven positive impacts of early intervention, the average age of ASD diagnosis in the U.S. is still very late, at around 4 years of age [2]. Identifying ASD in children who are deaf and hard of hearing (D/HH) can be particularly complex because some observable behaviors associated with autism may overlap with behaviors shaped by the experience of deafness. Diagnostic overshadowing occurs when professionals attribute a child’s behaviors or developmental differences primarily to a known diagnosis or condition [3] such as hearing loss, and consequently fail to recognize a co‐occurring condition, such as ASD. In the context of D/HH and ASD, diagnostic overshadowing may involve misattributing social‐communication differences, sensory responses, or unusual behaviors to hearing status alone, as well as limited familiarity among hearing clinicians with deaf culture and signed language development. It may also reflect insufficient attention to how hearing level, language access, cultural identification, communication modality, and family or social experiences shape the presentation of autism‐related characteristics in D/HH children [4, 5]. These factors can lead to delay in diagnosis and missed opportunities for D/HH children with ASD to benefit from life‐changing early intervention [5]. For an overview of developmental features commonly observed among children who are D/HH and characteristics consistent with ASD in this population, readers are referred to Szarkowski and colleagues [4].

We present a case of an adolescent male diagnosed with profound hearing loss at birth who demonstrated many behavioral and neurodevelopmental symptoms suggestive of autism yet was not referred for an autism assessment until the age of 13. This case highlights the challenges of assessing for neurodevelopmental disabilities in the D/HH population. “Red‐flag” indicators for the possible presence of autism in D/HH individuals will be described, as well as strategies for adapting autism assessment tools to this population. Finally, the importance of listening to family concerns to identify and treat autism early will be discussed. This case report follows the CARE Guidelines [6].

### 1.1. Patient Information

A 13‐year‐old adolescent male was referred for an interdisciplinary autism assessment. He exhibited a significant global developmental delay with minimal communication in any modality. His past medical history was complex and included traumatic delivery at birth (with a drop in heartrate and nuchal cord), neonatal hyperbilirubinemia, diminished bile ducts on liver biopsy at 1 month of age, diagnosis of congenital bilateral sensorineural hearing loss, and seizure disorder. He received pediatric neurology care for a seizure disorder until approximately 10 years of age, at which time his seizures resolved. He had not been on any antiepileptic medication since that time.

The patient received cochlear implants at the age of 2 but was unable to tolerate them. Specifically, any time the cochlear implants were activated, he would become emotionally distressed and remove them. Consequently, the patient never acquired a spoken language. In addition to being unable to acquire spoken language, the patient demonstrated significant deficits in joint attention, which reduced his ability to attend to social partners and reciprocate nonverbal communication. Because his attention to social partners was inherently limited, sign language was also not acquired. Shield and colleagues found that autistic children demonstrate minimal sign language acquisition despite full access to ASL from native adult signers [7]. In light of these findings and the patient’s social communication limitations, early, intensive speech–language intervention incorporating a robust, high‐tech augmentative and alternative communication (AAC) device may have better supported functional communication development. The patient was exposed to low‐tech AAC using a picture exchange communication system (PECS) and began intervention with a high‐tech, speech‐generating AAC device at the age of 10.

Early intervention records reported concerns for a “vision impairment” based on the observation that he displayed limited eye contact and used his vision in atypical ways. However, the patient received a subsequent formal vision exam that ruled out visual deficits. At the age of 3 years, the patient began attending developmental preschool through his local school district based on the special education eligibility category of global developmental delay. In the fourth grade, his eligibility classification was changed to multiple impairment to reflect dual diagnoses of intellectual disability and hearing impairment. Despite many reported behavioral concerns such as lack of social and peer engagement, sensory dysregulation, and restricted and repetitive behaviors, an autism spectrum diagnosis was never considered as these behaviors were attributed solely to his hearing loss.

At the age of 13, the patient was assessed by an interdisciplinary autism assessment team. Based on both history as well as examination using standardized, evidence‐based assessment tools, the patient met full criteria for ASD. In particular, he had no meaningful use of spoken language or nonverbal gestures. He showed little interest in other people and preferred to spend hours by himself on an iPad. With the disruption of routines, he exploded into distressed meltdowns. He also exhibited many of the classic behaviors of autism, such as complex finger movements, peering at objects at an angle, and hypersensitivity to visual stimuli. He scored in the ASD range on the Autism Diagnostic Interview‐Revised Deaf Adaptation (ADI‐R) (Table 1), Autism Diagnostic Observation Schedule, Second Edition Deaf Adaptation (ADOS‐2), Module 1 (Table 2), Childhood Autism Rating Scale, Second Edition (CARS‐2) (Table 3) and met full DSM‐5 criteria for ASD. The evaluating team rated him as needing very substantial support for severe deficits in both social communication and restricted and repetitive patterns of behavior.

**Table 1:** Results of autism diagnostic interview‐review (ADI‐R).

Autism diagnostic interview‐revised (ADI‐R)		
Domain	Score	Diagnostic cutoff
Qualitative abnormalities in reciprocal social interaction	28	10
Qualitative abnormalities in communication (verbal)	—	8
Qualitative abnormalities in communication (nonverbal)	14	7
Restricted, repetitive, and stereotyped patterns of behavior	10	3
Abnormality of development evident at or before 36 months	5	1

**Table 2:** Results of the autism diagnostic observation schedule, second edition module 1.

Module 1		
Social affect (SA)		
Communication		Few to no words
Frequency of spontaneous vocalizations directed to others	A‐2	2
Pointing	A‐7	—
Gestures	A‐8	2
Reciprocal social interaction		
Unusual eye contact	B‐1	2
Facial expressions directed to others	B‐3	2
Integration of gaze and other behaviors during social overtures	B‐4	2
Shared enjoyment in interaction	B‐5	2
Showing	B‐9	2
Spontaneous initiation of joint attention	B‐10	2
Response to joint attention	B‐11	2
Quality of social overtures	B‐12	2
SA total		20
Restricted and repetitive behaviors		
Intonation of vocalizations or verbalizations	A‐3	0
Stereotyped/idiosyncratic use of words or phrases	A‐5	—
Unusual sensory interests in play/material/person	D‐1	2
Hand and finger and other complex mannerisms	D‐2	2
Unusually repetitive interests or stereotyped behaviors	D‐4	2
RRB total		6
Overall total		26
Cutoff for autism		>16
Comparison score		10 (high)

**Table 3:** Childhood autism rating scale, second edition (CARS‐2) total score and interpretation.

Total raw score	48.5
T‐score	64
Percentile rank	92
Severity group	Severe symptoms of autism spectrum disorder (35 and higher for ages 13+)

### 1.2. Patient/Family Perspective

The author interviewed the patient’s primary caregiver to obtain information regarding the patient and family’s lived experience with the diagnostic process. The family discovered that the patient was deaf quickly after birth from the NICU staff. They were referred to early intervention services but despite treatment, felt that they did not see much developmental progress. The parents questioned whether something else could be present, but all medical professionals said that the patient’s behavior was due to him being deaf. A number of years ensued until he received an assessment and subsequent autism diagnosis at age 13. The patient’s parent reflected, “Being a younger mom at the time, I felt ignored and abandoned by the medical system. We were not provided any resources. The medical system needs to do better. I’m in this for life. I need help while I’m here, and support for him after I’m gone.”

### 1.3. Provider Perspective

The special education teacher in the patient’s self‐contained rural school classroom reported that the family had expressed concerns that their son may have an additional undiagnosed condition, such as autism, contributing to his challenges. However, school staff lacked knowledge and experience in this area. After pursuing additional training in autism through her state’s Leadership Education in Neurodevelopmental and Related Disabilities (LEND) training, a federally funded program that prepares healthcare and education professionals to support individuals with neurodevelopmental disorders [8], she referred the patient to a university‐based center specializing in complex autism evaluations. As a result of this case, the teacher’s rural school district has since engaged in further autism‐specific training and has improved its internal protocols to support earlier identification and treatment.

## 2. Discussion

This case highlights the importance of understanding and identifying the differences between hearing loss‐related behaviors and signs of ASD. In particular, a child who demonstrates ongoing language and communication delays despite interventions for hearing loss should be considered for possible co‐occurring ASD [5]. Similarly, deficits in reciprocal social communication such as poor eye contact, reduced use of gestures, lack of give/show behaviors and shared enjoyment, absence of joint attention, and problems with turn‐taking are not typical of D/HH and can be a warning sign or “red flag” of possible ASD [4]. Children with ASD may also have sensory sensitivities that make it difficult to tolerate cochlear implants, which is an additional “red flag” for possible ASD.

The adolescent boy in this case had many early signs of autism that were missed by both educational and health care professionals. Despite early intervention services and multiple inpatient stays in out‐of‐state urban academic medical centers that the family was referred to due to the lack of specialty care in their rural community, autism was not considered because his problematic behaviors and lack of language progress were attributed to hearing loss. At the age of 2 years, the activation of cochlear implants triggered severe emotional meltdowns, which in retrospect likely reflected sensory sensitivity. His limited eye contact during early intervention services was misattributed to visual impairment. While parents expressed concerns about his lack of social engagement with family and peers, as well as his rigid inflexibility and perseverative behaviors, the professionals continued to maintain that these concerns were explained by the hearing loss. Together, these factors suggest that residence in a rural area may have reduced access to ongoing specialized services and locally available expertise, thereby compounding diagnostic delays by limiting continuity of care and provider familiarity with complex neurodevelopmental presentations.

The interdisciplinary autism assessment team that evaluated this patient was careful to use standardized autism assessment tools that have been adapted for use with D/HH individuals. The gold‐standard ADOS‐2 has an adaptation for use with D/HH children and young people [9, 10]. Major adaptations in this tool include allowing the D/HH individual to communicate using their preferred language (sign or spoken), cultural adaptations of specific tasks (for example, waving in front of the child’s face and using their sign name or finger‐spelling their name to gain their attention, instead of calling out the child’s name from behind during the “response to name” task), and changes in coding to account for differences in the way that ASD symptoms may present in D/HH children (for example, idiosyncratic language in a D/HH child with autism might present as a child reversing the usual direction of a sign). The ADI‐R has also been adapted for use with deaf children and young people [11]. For future practice, practitioners may consider incorporating the Behavior Assessment System for Children, Third Edition (BASC‐3), a parent‐ and teacher‐report assessment tool, as part of a comprehensive evaluation, as one research group found that it successfully differentiated children with co‐occurring D/HH and ASD from children with D/HH alone [12].

A critical lesson of this case is the importance of listening to parents when they express concerns. Parents of children with disabilities are experts on both their unique child and their child’s condition [13]. In this case, the parents noticed their child’s social difficulties from early development, but they felt dismissed by professionals who insisted that their son’s hearing loss was the only explanation for his unusual behaviors. Studies show that early childhood interventions for autism significantly improve long‐term outcomes [14]. Ensuring that autism in children with D/HH is identified early will enable these children to access autism‐specific interventions (such as applied behavior analysis and speech language therapy) that will enhance their overall prognosis.

## 3. Conclusion

This case underscores the need for timely referral, interdisciplinary collaboration, and active follow‐through when early developmental concerns are identified in children who are D/HH. Although ASD can be reliably diagnosed by age 2, the average age of diagnosis remains above 4 years, highlighting the continued need to move beyond a “wait‐and‐see” approach. Families should be supported through prompt referrals, care coordination, education about their child’s developmental profile, and guidance regarding their role in intervention. For children with significant social communication needs, early intervention should include access to intensive, individualized speech‐language services and consideration of a high‐tech AAC when appropriate. Training programs for educational and clinical professionals should also strengthen coursework in neurodevelopmental disorders and dual diagnoses to improve early identification, referral pathways, and collaborative care across medical and educational systems.

## Author Contributions

Stephanie E. King conceived the study, collected the data, wrote portions of the manuscript, and reviewed and edited the final draft. Erika Ryst collected the data, wrote portions of the manuscript, and reviewed and edited the final draft. Dr. Stephanie King and Dr. Erika Ryst collected data and contributed to the drafting, editing, and revision of the manuscript.

## Funding

The authors received no financial support for the research or authorship of this article. Partial funding for open access was provided by the University of Nevada, Reno School of Medicine Open Access Fund.

## Disclosure

All authors approved the final manuscript as submitted and agree to be accountable for all aspects of the work. Stephanie E. King and Erika Ryst met the ICMJE authorship criteria.

## Ethics Statement

In accordance with local institutional policies, Institutional Review Board (IRB) approval was not required because this study was a retrospective case report and did not involve any experimental intervention.

## Consent

Written informed consent was obtained from the patient’s legal guardian for the collection and publication of this clinical case and any accompanying data. Every effort has been made to protect patient anonymity.

## Conflicts of Interest

Stephanie E. King serves as the director of the University Center for Autism and Neurodevelopment (UCAN) at the University of Nevada, Reno. The other author declares no conflicts of interest.

## Data Availability

The data that support the findings of this study are available upon request from the corresponding author. The data are not publicly available due to privacy or ethical restrictions.
